# Understanding the attitudes of the elderly towards enrolment into cancer clinical trials

**DOI:** 10.1186/1471-2407-6-34

**Published:** 2006-02-08

**Authors:** Carol A Townsley, Kelvin K Chan, Gregory R Pond, Christine Marquez, Lillian L Siu, Sharon E Straus

**Affiliations:** 1 Princess Margaret Hospital (PMH) and University of Toronto, Toronto, Canada; 2 Toronto General Hospital and University of Toronto, Toronto, Canada

## Abstract

**Background:**

The optimal cancer treatment for an older population is largely unknown because of the low numbers of elderly patients accrued into clinical trials. This project focuses on the attitudes of the elderly about participation in clinical trials to determine if this is one of the barriers to the involvement of this population in clinical trials.

**Methods:**

The first phase of this study was a self-administered questionnaire mailed to 425 elderly persons with cancer, selected from Princess Margaret Hospital oncology clinics. The second phase consisted of individual semi-structured interviews with cancer patients to assess their attitudes towards cancer, its management and enrolment into cancer clinical trials.

**Results:**

Ninety-four patients responded to the survey giving a response rate of 22.1%. Three quarters of respondents stated that they would be willing to participate in a clinical trial. The factors that most influenced older patients' willingness to participate in a cancer study were recommendations from a cancer doctor and the chance that the study treatment may help them feel better. Seventeen survey responders participated in interviews. Common themes from these interviews included patient-physician communication, the referral process, and the role of age in cancer care decision-making.

**Conclusion:**

Most elderly people, who responded to this survey, are willing to consider participation in cancer clinical trials however, elderly patients do not appear to actively seek clinical trials and few were informed of the availability of clinical trials. Physician barriers and availability of appropriate clinical trials may play a bigger role in preventing accrual of elderly cancer patients into trials.

## Background

Cancer is a disease of the elderly with 60 % of cancers occurring in those over the age of 65 years [1]. As our population ages it will become increasingly critical to optimise treatment in older patients to ensure both good quality of life for this group and the best allocation of medical resources. The treatment of cancer in the older patient is challenging for a multitude of reasons. Firstly, care of the older patient is often complicated by comorbidities and other physiological factors, particularly deteriorating renal, bone marrow, and metabolic reserve [2]. This makes it extremely difficult to extrapolate information concerning cancer care based on a younger population to older patients. Secondly, there is a lack of participation of elderly cancer patients in clinical trials thus limiting the available information for physicians on the appropriate management for this group [3-5]. Many elderly patients with other co-morbid conditions may not be accrued into clinical trials because of protocol eligibility requirements. Lastly, as with all clinicians, the cancer specialist is frequently faced with situations where they must balance their personal beliefs, professional values, and knowledge of medicine with their patients' preferences and needs [6].

There have been many studies examining the lack of accrual of elderly patients into clinical trials both in the United States and in Canada. For instance, a study examining the data on over 16000 patients consecutively enrolled between 1993 and 1996 in 164 studies conducted by the Southwest Oncology Group (SWOG) in the U.S. found that patients aged 65 years or older accounted for only 25% of patients enrolled into the trials even though they accounted for more than 60% of the U.S. population with cancer [7]. A study in Canada demonstrated similar results. While persons aged 65 or older account for 58% of the Canadian population with cancer, they were only enrolled into studies conducted by the National Institute of Canada (NCIC) at a rate of 22% [8]. A recent review of the barriers to the recruitment of older patients with cancer onto clinical trials suggested that age continues to be a significant barrier to recruitment [9]. It appears that physician's perceptions of the elderly, strict protocol eligibility criteria, and restrictions on enrolling patients with comorbid conditions may play the biggest roles in preventing the accrual of older patients. Unfortunately, although physician barriers, protocol barriers and logistic barriers have been examined, there is a lack of research investigating the older patient's attitude towards clinical trials.

In order to gain a better understanding of possible causes for the low accrual rate of older patients with cancer into clinical trials the patient's perspective must be explored. This study addresses the understanding and the attitudes of the elderly towards enrolment into cancer clinical trials. We have attempted to determine whether patient's awareness and understanding of clinical trials, play a role in the low accrual rate of elderly patients into cancer clinical trials.

## Methods

This project was performed in two phases.

### Phase 1: Mailed survey

An invitation to participate in a self-administered questionnaire to identify and understand attitudes of the elderly towards enrolment into cancer clinical trials was mailed to 425 elderly persons with cancer. All patients aged 70 and older who were seen at Princess Margaret Hospital (PMH) oncology follow-up GI, lung and breast clinics in April 2003 were invited to participate. One hundred and fifty four peoplestated willingness to participate and they were subsequently sent the questionnaire. Although patients were recruited mainly from GI, breast and lung clinics, patients from ambulatory clinics of other tumor types were also approached. Also, patients with two primary tumors were eligible and thus additional types of cancers could be represented. There were no requirements for active treatment or previous or ongoing clinical trial participation. Participants had to have a life expectancy of more than 12 weeks and to be able to read English.

Mailed reminders were sent at two and four weeks to maximise the survey response rate. The survey was completed anonymously and participants were instructed to avoid including their name or any other unique information on the survey. To encourage response, a stamped, addressed envelope and a brief letter describing the goals of the survey were included with every survey package. Participants were also asked to complete and return an addressed postcard if they were interested in participating in a one-on-one interview at a later date.

By completing an extensive literature review to identify barriers to cancer care (including enrolment in clinical trials) for older patients, we composed a comprehensive survey consisting of a large number of items. Using a modified Delphi technique, 4 investigators reduced the list to a final group of questions felt to be most crucial to identifying important issues for this patient population. Prior to administration, the survey was pilot-tested with a group of 5 elderly people to ensure that the survey was clear and had appropriate face validity.

### Data collection and analysis

Two members of the research team independently entered the results of the survey into a database. Summary statistics such as the median, percentage and frequency were used to describe the respondents. Three potential predictor variables, age, self-reported health status and whether the patient believed they were cured of cancer or not, were investigated for potential association with willingness to participate in and factors affecting decision to participate in clinical trials. To increase statistical power, categorization of patients into two groups was performed for age (< 75 versus ≥ 75) and health status (excellent/good versus poor/marginal/average). Similarly, level of agreement to attitudes towards clinical trial statements was dichotomized (strongly agree/agree versus strongly disagree/disagree/neither disagree nor agree). Potential associations were investigated using the chi-square test. All tests were two-sided and p-values of 0.05 or less were considered statistically significant with no adjustment to the p-value performed for multiple testing.

### Phase 2: One-on-one semi-structured interviews

#### Study design

One-hour semi-structured interviews were completed with those survey respondents who expressed interest in this phase. This interview was conducted to further explore their attitudes about cancer, cancer therapy, and participation in clinical trials. A research coordinator with extensive interview experience conducted the interview. All participants received a stipend of $30 to compensate for transportation and parking costs. Participants were encouraged to speak feely, to raise issues that were important to them and to support their responses with examples. Domains of inquiry were identified from discussion among the authors (CAT, KC, LLS, SES) after review of the results of the survey. The interview tapes were transcribed verbatim and assigned a unique identifier for each participant.

#### Analysis of interviews

Grounded theory was used to analyse the data from the interviews by generating categories and themes [10]. The analysis was initiated after the first interview to allow emerging themes to be explored in subsequent interviews. Sampling of participants continued until saturation was achieved and no new themes were identified. Two investigators, who were blinded to the identity of the participant, independently coded the data to increase reliability. After the transcripts were checked for accuracy the tapes were destroyed.

#### Ethics

Patients were informed that they could refuse to participate at any time and that this would not impact on their future care. Consent to participate in Phase 1 of the study was implied by return of the postcard and completion of survey. Participants were asked to complete a consent form prior to the interview. Data did not include any unique identifiers and none of the clinicians involved in the care of the patients had access to this data. Ethics approval was obtained from the University Health Network and the University of Toronto.

## Results

### Phase 1: Self-administered questionnaire mailed to elderly persons with cancer

#### Baseline demographics

Ninety-four questionnaires were returned resulting in a response rate of 61.4% (considering those that had originally agreed to participate) and 22.1% (considering the original cohort who were invited to participate). The demographics of the responders can be found in Table 1. Over 80% of responders were between the ages of 70 and 79, approximately half were female and most responders were living with a significant other. Over 50% of respondents rated their health as good or excellent, with only 2% feeling that their health was poor. When asked about clinical trials (table 2), most patients (70%) responded that they had never participated in a clinical trial and only 3% had ever sought information themselves about a clinical trial. Most elderly patients with cancer felt that the two most important treatment decision makers were themselves and their cancer or family doctor.

#### Attitudes towards clinical trial participation

Patients were asked three questions regarding their attitudes towards participating in a clinical trial (Table 3). About three quarters of patients would participate in clinical trials to prevent or screen for cancer, just over half would participate in clinical trials comparing a new drug to a 'standard' drug, and 70% would participate in clinical trials to test a new drug when there is no 'standard' drug available. The only statistical significant predictor of whether the patient would enrol in a clinical trial was whether the patient believed they were cured as a predictor of enrolment in a screening or prevention trial. Those patients who believed they were cured indicated they were less likely (62% versus 88%) to participate (p-value = 0.009).

#### Reasons to participate in a clinical trial

Respondents were asked about the reasons for participating in a hypothetical clinical trial (Table 4). The three highest-ranking answers were a recommendation from the cancer doctor, a chance that the patient may feel better because of the treatment and a chance that this study may help other cancer patients in the future. Interestingly, the chance that the patient may live longer was rated lower than the previously mentioned items, as only about 90% agreed that an increased life span would affect their decision to participate. Recommendation from the family doctor was felt to be important for decision making for about 80% of responders and recommendations from family/friends ranked last with less than 60% of responders feeling it was important. There was no significant difference in results based on age or health status. However, patients who were not cured felt that the chance that they may live longer was an important decision maker for them (p-value = 0.012).

#### Reasons to decline participate in a clinical trial

Respondents were asked about factors that would affect their decision not to participate in a clinical trial (Table 5). The two highest ranking reasons not to participate in a clinical trial were a recommendation from the cancer doctor against participating and concerns about whether or not the new treatment works. None of the answers differed significantly based on their age group, their health status or whether or not the felt they had been cured of the cancer. Recommendations from family, friends, or the family physician would not have a significant impact on the decision to enter a clinical trial. Similarly, age was not seen as a reason for not participating in a clinical trial. No statistically significant associations between age, health status or perceived curative status was observed with any factor.

### Phase 2: Semi-structured one-on-one interviews with older cancer patients

Seventeen survey respondents agreed to participate in interviews. Baseline demographics can be found in table 6. Most respondents were between the ages of 70 and 74, with only one older than 85 years. More than half of the respondents were male, most lived on their own at home, many had completed post-secondary education and the most common tumor type was colorectal cancer. Most patients rated their health status as above average. Several themes were identified around the attitudes that the patients had towards cancer care and clinical trials including; patient-physician communication, the referral process, consent to clinical trial participation, the randomization process and the role of age in cancer care decision making.

#### Patient-physician communication

In all areas of medicine it is essential to have good communication between the patient and the physician. Most participants felt that the initial communication regarding diagnosis was well done and although over half were alone when they received news of their diagnosis, most felt they would not have preferred to have someone there with them at this moment. Patients stated that the most important factor in feeling comfortable, while getting the initial diagnosis, was confidence in the physician giving the news. One responder stated; "I think the key is that he was young, vigorous man, who communicates very well and is able to tell the patient exactly what the score is in every respect, the techniques involved in surgery, the chances of success or otherwise, and as I've said, he instils confidence in the patient..."

Twelve participants felt they were happy to have had someone accompany them when discussing cancer management options as this person was able to ask more questions and listen to information as well as to lend support. All participants felt they had made final decisions regarding cancer care themselves based on recommendations from the cancer specialist. However, the treating physicians heavily influenced some participants' treatment decisions. One responder stated; "I would choose whatever had been recommended to me by the doctor or doctors...I think that applies to pretty well everything..."

#### The referral process

Almost all respondents expressed a desire that the time to removal of the primary tumour be as quick as possible if this was an option in their treatment plan. According to the patients waiting times ranged from 1 to 4 weeks from diagnosis to surgery or referral to a radiation or medical oncologist. None of the respondents felt that they had waited an unacceptable amount of time but almost all expressed a desire to be treated quickly.

#### Consent to clinical trial participation

There appeared to be some misunderstandings about clinical trials and what participation would entail. For example, many participants felt that the treating physician should decide which treatment was better for them in a clinical trial and that the patient should then receive that arm. One patient stated he had never heard the expression "clinical trial". Only 2 patients had been approached to participate in a clinical trial excluding our study. Most interview respondents felt they would be happy to consider participating in a clinical trial if there was one available for their particular situation. None of the 15 patients who had not been approached about a clinical trial were aware of any information about clinical trials available from their physician or another source. When asked about the best way to communicate information about clinical trials to elderly patients with cancer, many respondents felt that using a newsletter sent to patients would be appropriate. Some also felt that this information should come from the treating physician. One patient stated, "maybe if there was a newsletter type thing sent to doctors that could be put in their office, detailing some of the benefits of this, it might help generally to get people thinking in the right direction."

#### The role of patient age in cancer care decision-making

All respondents felt that age should not be a factor if there was no limitation on the amount of resources available. One respondent stated: "You could have a much younger person in poor health and you could have an older person in good health, it (treatment) should depend on each particular individual not their age." If resources were limited some felt that age should be taken into account. One responder felt the decision not to perform surgery had been based on his advanced age but at the same time was glad that he did not go through an invasive procedure.

## Discussion

Although there have been studies looking at the patient's perspective regarding their cancer and treatment, there is a paucity of information about how elderly patients feel about clinical trials. As well, much of the available research evaluates the patient's attitude towards the screening of different types of cancer and not at their overall treatment. The elderly population experiences different care from their younger counterparts as they are treated less aggressively and enrolled in clinical trials less often. The lack of data on the management of elderly patients with cancer and their risk of adverse events with therapy make management decisions difficult. Having the general knowledge of the patient's beliefs may help to guide the clinician regarding the best management approach.

This study takes an in-depth look at the attitudes of elderly patients with cancer towards their disease and clinical trials in cancer care. Two different methods were used to address these issues; a mail out survey and one-on-one interviews. Interestingly, the cancer specialist continues to be a very important figure when deciding to participate in a clinical trial. Surprisingly, the chance of increased survival was less important than the chance for better quality of life when ranking reasons to participate in a clinical trial. The influence of family and friends did not appear to be very important in making decisions about participation in a clinical trial. Patients who were alone when they met with their physicians at the time of their diagnosis felt they did not need other family or friends there and relied more on the physician's opinion.

There have been a few other studies looking at elderly cancer patient's attitudes towards cancer care [11-16]. Most studies demonstrated that elderly patients had very similar views as their younger counterparts concerning issues of cancer screening and cancer care. A study by Yellen et al. looked at age and clinical decision-making in oncology patients using clinical vignettes in an interview situation. They found that older patients were as likely as their younger counterparts to agree to chemotherapy for both curative and control purposes, yet the older patients were less willing to accept a more toxic treatment for an increase in survival [17]. This is in keeping to the trends we observed, showing that older patients placed slightly less importance on the chance of living longer as a reason to participate in a clinical trial. A much higher proportion of those who stated they had not been cured of their disease felt that the increased chance of survival was a very important factor when making a decision to participate in a clinical trial. We might hypothesize that those not cured of their disease feel much more vulnerable with regards to their own mortality and therefore would place more value on any extra survival time, regardless of quality of life.

There has been one study examining how often elderly breast cancer patients were offered clinical trials and their attitudes towards them [18]. They found that sixty-eight percent of younger stage II patients were offered a trial compared with 34% of the older patients and of those offered a trial, there was no significant difference in participation between younger (56%) and older (50%) patients. These trends were seen with our patient population as well where few older patients had been offered a clinical trial but the majority would be willing to participate in one.

Although this study draws attention to many important points regarding the attitudes of the elderly patients with respect to care of their cancer and clinical trial participation, there are a few limitations to this study. Our response rate, was lower than we would have preferred but our results were based on almost 100 elderly patients representing one of the largest series of elderly cancer patient surveys. It was our intention to target a well elderly population to compare their responses with those from patients with cancer. However, this was difficult due to the lack of healthy control participants in the hospital and the reluctance of the ethics review board to approve the targeting of a random external population. This low response rate does present the potential for response bias. It is possible that the patients who agreed to participate in this survey are also the ones that would be more inclined to participate in clinical trials by their very nature. This may make it difficult to extrapolate these results to the general elderly population who may have a different view on clinical trials. This study however, is still the largest cohort of elderly cancer patients to be asked about clinical trials and the results are interesting. Clearly more research will need to be done on a larger population in the future.

One of the strengths of this study was the addition of individual interviews to explore patient's perceptions of participation in clinical trials. When performing qualitative research such as the interpretation of individual interviews, efforts must be made to establish methodological rigour and to avoid potential bias. Our team tried to minimize this by having more than one member independently analyze the data and develop the framework of themes that arose from the results.

Other factors may play a role in affecting the accrual of elderly patients onto clinical trials including the higher prevalence of co-morbid conditions in the elderly which in some instances may make them ineligible for studies and at times logistic problems such as more difficulty traveling to centres where clinical trials are being performed. Our group did evaluate the referral patterns of Ontario primary care physicians and found that although 86% of respondents would refer most older patients with early-stage, potentially curable cancers to oncologists, only 65% would refer those with advanced-stage, potentially incurable cancers [18]. This may influence the type of patients recruited to this study as some elderly patients with cancer are not being referred to a cancer centre and therefore do not have the opportunity to consider a clinical trial.

It would appear that most older patients with cancer, who responded to this survey, would willingly consider participation in a clinical trial. However few older cancer patients were informed of the availability of clinical trials nor were they active in seeking the availability of clinical trials. This may in part be due to the lack of knowledge in this population concerning clinical trials in general. Physician barriers may play a bigger role in preventing accrual of elderly cancer patients onto trials.

## Conclusion

Most elderly people, who responded to this survey, are willing to consider participation in cancer clinical trials however, elderly patients do not appear to actively seek clinical trials and few were informed of the availability of clinical trials. Physician barriers and availability of appropriate clinical trials may play a bigger role in preventing accrual of elderly cancer patients into trials.

## Competing interests

The author(s) declare they have no competing interests

## Authors' contributions

CT: Study development, review of data and manuscript writing

KC: Study development, collection of data and manuscript writing

GP: Statistical analysis of data and manuscript review

CM: Collection of data and manuscript review

LS: Study development, review of data and manuscript review

SS: Principal investigator, study development, review of data and manuscript review

## Pre-publication history

The pre-publication history for this paper can be accessed here:



## Figures and Tables

**Table 1:** Participant demographics of mailed survey (n = 94)

**Variable**	**# respondents (% respondents)**
Age	
70–74	46 (49)
75–79	33 (35)
80–84	14 (15)
85–90	1 (1)
> 90	0 (0)

Gender:	
NA	1 (1)
Male	51 (54)
Female	42 (45)

Living situation*	
By yourself?	29 (31)
With spouse/significant other?	64 (68)
In a retirement home?	2 (2)
With children?	2 (2)
With friends?	0 (0)
In a nursing home?	0 (0)

Current health status?	
NA	3 (3)
Poor	2 (2)
Marginal	13 (14)
Average	28 (30)
Good	40 (43)
Excellent	8 (9)

Type of cancer	
Skin (not melanoma)	13 (14)
Melanoma	4 (4)
Lung	5 (5)
Breast	17 (18)
Prostate	19 (20)
Colon/rectum	27 (29)
Lymphoma	8 (9)
Other	29 (31)

Median (Range) Age at diagnosis (n = 91)	68 (41–81)

To the best of your knowledge, are you considered cured of cancer?	
NA	3 (3)
Yes	31 (33)
No, but I am currently receiving treatment	27 (29)
No, but I was previously treated	20 (21)
No, I have never had any treatment	2 (2)
Do not know	11 (12)

* Multiple answers were possible

**Table 2:** Clinical trial demographics from mail survey

**Clinical trial demographics**	**# respondents (% respondents)**
Participation in a cancer clinical trial	
Yes	22 (23)
No	70 (74)
NA	2 (2)

Invited to participate in a cancer clinical trial n = 69:	
Yes	13 (19)
No	56 (81)

Sought information about a cancer clinical trial n = 67	
Yes	2 (3)
No	65 (97)

Participants in decision making	
Yourself	73 (78)
Family	42 (45)
Friends	4 (4)
Your cancer/family doctor	81 (86)
Others	6 (6)

**Table 3:** Clinical trial participationNumber (%) of patients who agree/strongly agree to survey questions grouped by demographic characteristics of interest

	**Age**		**Health Status**		**Cancer Status**	
**Question**	n/N (%) < 75 n/N (%) ≥ 75	p-value	n/N (%) Good/Excellent n/N (%) Poor/Marginal/Ave	p-value	n/N (%) Cured n/N (%) Not Cured	p-value

1) I would participate in 'clinical trials' that study ways to prevent or screen for cancer	34/44 (77) 35/46 (76)		36/47 (77) 30/40 (75)		18/29 (62) 42/48 (88)	
		0.89		0.86		0.009
2) I would participate in a 'clinical trial' to compare a new drug to the old "standard" drug	24/43 (56) 21/46 (46)		24/47 (51) 20/39 (51)		13/30 (43) 25/47 (53)	
		0.34		0.98		0.40
3) I would participate in a clinical trial to test a new drug when there is no "standard" drug available	34/45 (76) 30/47 (64)		32/47 (68) 29/42 (69)		18/30 (60) 37/49 (76)	
		0.22		0.92		0.15

**Table 4:** What factors are important in your decision to participate in a clinical trialNumber (%) of patients who agree/strongly agree to survey questions grouped by demographic characteristics of interest

	**Age**		**Health Status**		**Cancer Status**	
**Question**	n/N (%) < 75 n/N (%) ≥ 75	p-value	n/N (%) Good/Excellent n/N (%) Poor/Marginal/Ave	p-value	n/N (%) Cured n/N (%) Not Cured	p-value

Recommendation from cancer doctor to participate	45/46 (98) 44/47 (94)		45/47 (96) 41/43 (95)		30/31 (97) 46/49 (94)	
		0.32		0.93		0.56
Recommendation from the family doctor to participate	36/46 (78) 40/46 (87)		38/47 (81) 35/42 (83)		25/31 (81) 39/48 (81)	
		0.27		0.76		0.95
Recommendation from family/friends to participate	26/46 (57) 26/45 (58)		24/49 (49) 23/39 (59)		17/31 (55) 28/47 (60)	
		0.90		0.35		0.68
The chance that you may live longer because of the treatment	43/46 (93) 40/46 (87)		41/47 (87) 39/42 (93)		24/31 (77) 46/48 (96)	
		0.29		0.38		**0.012**
The chance that you may feel better because of the treatment	44/46 (96) 44/46 (96)		45/47 (96) 40/42 (95)		30/31 (97) 45/48 (94)	
		1.00		0.91		0.55
The chance that this study may help other cancer patients in the future	44/45 (98) 43/46 (93)		44/46 (96) 40/42 (95)		30/31 (97) 44/47 (94)	
		0.32		0.93		0.54
Your age	31/44 (70) 28/46 (61)		29/45 (64) 28/42 (67)		20/30 (67) 29/47 (62)	
		0.34		0.83		0.66

**Table 5:** What factors are important in your decision NOT to participate in a clinical trialNumber (%) of patients who agree/strongly agree to survey questions grouped by demographic characteristics of interest

	**Age**		**Health Status**		**Cancer Status**	
**Question**	n/N (%) <75 n/N (%) ≥ 75	p-value	n/N (%) Good/Excellent n/N (%) Poor/Marginal/Ave	p-value		p-value

Recommendation from cancer doctor against participating	41/44 (93) 44/46 (96)		43/46 (93) 39/41 (95)		28/30 (93) 44/47 (94)	
		0.61		0.74		0.96
Your cancer doctor did not give you any advice about participating	34/42 (81) 38/45 (84)		34/44 (77) 35/40 (88)		23/28 (82) 37/46 (80)	
		0.67		0.22		0.86
Recommendation from your family doctor against participating	34/44 (77) 38/46 (83)		39/47 (83) 30/40 (75)		25/30 (83) 35/47 (74)	
		0.53		0.36		0.36
Your family doctor did not give you any advice about participating	22/43 (51) 28/45 (62)		24/45 (53) 24/40 (60)		16/28 (57) 25/47 (53)	
		0.30		0.54		0.74
Recommendations from your family and friends against participating	17/44 (39) 22/45 (49)		19/47 (40) 17/39 (35)		12/30 (40) 20/46 (43)	
		0.33		0.77		0.76
Your age	27/43 (63) 26/46 (57)		26/46 (57) 25/40 (63)		16/29 (55) 27/47 (57)	
		0.55		0.57		0.85
You have other health problems that make you feel too weak to participate	34/44 (77) 32/43 (74)		31/44 (70) 32/40 (80)		20/29 (69) 35/46 (76)	
		0.76		0.31		0.50
You have concerns about whether or not the new treatment works	41/44 (93) 40/46 (87)		40/46 (87) 38/41 (93)		27/30 (90) 41/47 (87)	
		0.33		0.38		0.71
You are afraid of possible side effects from the new treatment	33/44 (75) 40/46 (87)		34/46 (74) 36/41 (88)		22/30 (73) 38/47 (81)	
		0.15		0.10		0.44

**Table 6:** Participant demographics of one-on-one interview participants (n = 17)

**Variable**	**# respondents (% respondents)**
Age	**# respondents (% respondents)**
70–74	11 (65)
75–79	4 (23)
80–84	1 (6)
85–90	1 (6)
> 90	0 (0)

Gender:	
Male	9 (53)
Female	8 (47)

Education Level	
Completed post secondary education	4 (23)
Completed High School	8 (47)
Completed Primary School	5 (30)

Participant's perception of health status	
Poor	0
Marginal	1 (6)
Average	6 (35)
Good	4 (24)
Excellent	6 (35)

Living situation*	
By yourself?	11 (65)
With spouse/significant other?	6 (35)
In a retirement home?	2 (12)
With children?	2 (12)
With friends?	0 (0)
In a nursing home?	0 (0)

Type of cancer	
Skin (not melanoma)	2 (12)
Melanoma	1 (6)
Lung	1 (6)
Breast	2 (12)
Prostate	3 (17)
Colon/rectum	6 (35)
Lymphoma	2 (12)
